# Anthocyanins from fruit and vegetable waste: biosynthesis, extraction, and gut health benefits

**DOI:** 10.1016/j.fochx.2026.103614

**Published:** 2026-02-02

**Authors:** Hudda Ayub, Husnat Ahmad, Syeda Hijab Zehra, Khadija Ramzan, Muhammad Adnan Arif, Naima Tariq, Maria Teresa Capucchio, Robert Mugabi, Aanchal Sharma, Gulzar Ahmad Nayik

**Affiliations:** a National Institute of Food Science & Technology, University of Agriculture, Faisalabad, Pakistan; b Lithuanian Research Centre for Agriculture and Forestry, (LAMMC), Babtai, Lithuania; c Department of Veterinary Sciences - Pathology Unit, University of Turin, Italy; d Department of Food science and technology, Faculty of Food Science and Nutrition, Bahauddin Zakariya University, Multan, Pakistan; e Department of Food Technology and Nutrition, Makerere University, Kampala, Uganda; f University Centre for Research and Development, Chandigarh University, Mohali 140413, Punjab, India; g Marwadi University Research Centre, Department of Microbiology, Marwadi University, Rajkot 360003, Gujarat, India

**Keywords:** Anthocyanins, waste valorization, extraction, purification, quantification, stabilization, food applications, Gut health, health benefits

## Abstract

Anthocyanins (ANCs), naturally abundant in fruit and vegetable waste, represent a promising class of sustainable functional ingredients with notable gut-related health benefits. This review uniquely focuses on waste-derived anthocyanins and their microbiome-modulating effects, including enhancement of intestinal barrier integrity, prebiotic-like activity, and short-chain fatty acid production. Advances in green extraction and purification techniques such as ultrasound-, microwave-, enzyme-assisted, pulsed electric field, and deep eutectic solvent-based methods have improved recovery efficiency while promoting environmental sustainability. Furthermore, stabilization strategies including acylation, protein/polysaccharide complexation, co-pigmentation, and nano/microencapsulation enhance anthocyanin bioavailability and industrial applicability. By integrating technological innovation with gut health insights, this review highlights emerging opportunities to valorize agro-industrial waste and develop next-generation nutraceutical and functional food systems.

## Highlights

• Anthocyanins recovered from fruit and vegetable processing waste.
• Traditional and green extractions influence anthocyanin yield, purity.
• Purification, quantification, and stabilization ensure anthocyanin integrity.
• Applications include food colorants, functional additives, and nutraceuticals.
• Anthocyanins promote gut health and protect against chronic diseases.

## 1 Introduction

Ongoing trends in population growth and consumption levels are expected to continue, leading to a sustained need for increased food production over the next four decades (Barrera & Hertel, 2021). Meeting these demands will require a dual strategy involving the enhancement of food production capabilities, alongside efforts to reduce food losses and waste (Sarker et al., 2024a). Food loss and waste present environmental challenges, as well as social and financial issues, in both industrialized and underdeveloped countries (Amicarelli et al., 2021a; Jaiswal, 2023). The Food and Agriculture Organization (FAO) has provided estimates indicating that fruit and vegetable losses and waste amounts to approximately 60% of all horticultural output (Castillejo & Martínez-Zamora, 2024). In particular, by-products of fruit and vegetable processing contribute to 25-30% of this loss (Radha et al., 2024). Fruits, including grapefruits, oranges, apples, pineapples, chokeberries, and vegetables such as carrots, asparagus, potatoes, and onions, are subjected to processing to generate high-value derivatives (Kowalski & Gumul, 2024; Lucarini et al., 2021). Seeds, skins, pomace, and rinds are among the by-products of these processing procedures. Although these parts are normally not eaten, they do contain significant bioactive substances, including secondary metabolites and phytochemicals that remain trapped in the tissues (Fierascu et al., 2020a; Kumar et al., 2021a; Sha et al., 2023).

Agricultural and industrial wastes of fruits and vegetables include important chemicals called anthocyanins (ANC’s), which have attracted interest. Because of the difficult extraction procedure from waste, ANC’s are not used as often as they can be employed as additives in food processing, despite their substantial potential in technology, practicality, and economics. Therefore, choosing the extraction technique and establishing extraction parameters play pivotal roles in the recovery and purification of ANC’s for incorporation into food systems (Fierascu et al., 2020b). By improving current practices, it will be possible to recover more bioactive chemicals from food loss, thereby increasing their economic value in the feed and agri-food sectors. Approaches are necessary to address environmental concerns; raise consumer awareness about the substantial issue of food waste; and foster sensible, long-term, and equitable expansion (Byun et al., 2021; Jaiswal, 2023). The public has become more aware of these ideas in the past few years, as food waste has a major negative effect on the environment and availability of food worldwide (Huang et al., 2020).

ANC’s are water-soluble pigments that belong to a group of phenolic chemicals known as flavonoids. These compounds are glycosides of anthocyanidins, which are polyhydroxy and polymethoxy derivatives of the 2-phenylbenzopyrylium (flavylium) cation (Alam et al., 2021; Habtemariam, 2019). Two rings of aromatic compounds, designated A and B, are present in anthocyanidins and are divided into a heterocyclic ring with six members (C) that contain oxygen. The ANC molecules with positively electrically charged oxygen atoms impart hydrogen-donating antioxidant properties and impart a vivid reddish-orange to blue-violet color under acidic conditions. Typically, ANC molecules possess a carbohydrate group attached via esterification at the 3-position (Di Salvo et al., 2023). Xylose, galactose, rhamnose, arabinose, and glucose are the most frequently occurring sugars linked to anthocyanidins (Constantin & Istrati, 2022).

ANC’s, which include pigments that dissolve in water, impart distinctive blue, red and purple colors to a variety of vegetables and fruits such as pomegranates, cherries, grapes, onions, radishes, red cabbage, and more (Khoo et al., 2017). An increasing number of people are interested in learning more about the possible advantages of ANC’s in their diets, both in terms of their ability to prevent disease and as a means of promoting a healthy lifestyle. Although their association with cardiovascular disorders is where ANC’s show the most promise, they are frequently mentioned as exhibiting anti-inflammatory and antioxidant qualities in the scholarly literature (Wang et al., 1999).

ANC’s are typically produced by plants and play an essential role in imparting red, blue, and purple hues to an extensive variety of vegetables, fruits, and berries. Their presence in various plant parts, including vegetative organs, flowers, and fruits, has significant benefits for these plant species (Chen et al., 2019; Zhang, Celli, & Brooks, 2019). ANC’s found in plants can exhibit an extensive array of structural diversity, based on elements such as the quantity of hydroxyl groups, location of attachment of sugar units, type of sugar (frequently encountered ones being arabinose, galactose, glucose, rhamnose, and xylose), glycosidic linkage (α or β linkage), and complexity of the sugars (ranging from monosaccharides to disaccharides and trisaccharides) (Escalante-Aburto et al., 2023; Miladinovic et al., 2023; Yan, Wang, et al., 2020).

The review provides an overview of recent developments in the extraction, stabilization, quantification, and bioavailability of anthocyanins from fruit and vegetable by-products. Particular attention is given to: (i) emerging green extraction technologies that enhance recovery efficiency and sustainability; (ii) molecular stabilization strategies including acylation, co-pigmentation, and encapsulation; (iii) advanced analytical platforms such as LC–MS and QTOF–MS for high-precision characterization; and (iv) bioavailability enhancement approaches that enable the effective incorporation of anthocyanins into functional foods.

## 2 Extraction of anthocyanins

Although agro-industrial waste contains valuable organic materials that serve as reservoirs for various components, ensuring the safety of recovery, purification, and concentration of these bioactive molecules remains a challenge (Freitas et al., 2021). Factors such as the source of the waste, the activity of the molecules, their chemical characteristics, and the intended application all impact the extraction of bioactive compounds from industrial and agricultural wastes (Fang et al., 2022). Numerous methods are commonly utilized to extract bioactive chemicals from food residues, including CO_2_ extraction, solvent extraction, steam distillation, and Soxhlet extraction. Nevertheless, despite their convenience, affordability, and minimal equipment requirements, conventional approaches for extracting organic solvents have significant drawbacks (Dini, 2021). These include a limited selection of suitable solvents and the significant solvent quantities needed for extraction. Many of these solvents are volatile and toxic, and present safety concerns regarding human consumption and potential carcinogenic risks. In addition, conventional methods are associated with extended extraction periods and comparatively poor waste-derived bioactive chemical recovery rates (Wani et al., 2021).

**Fig 1:**
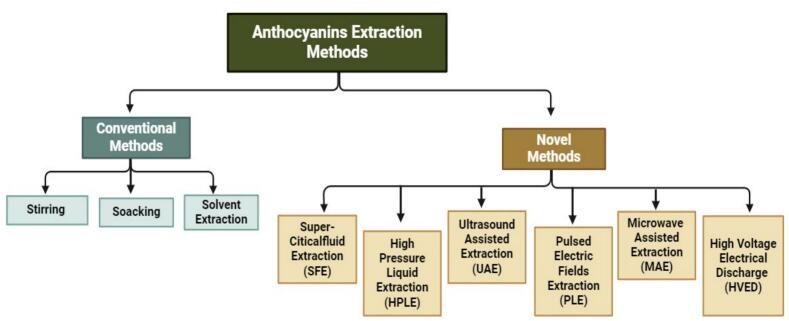
ANC’s systematic flow diagrams of extraction methods

These methods provide environment-friendly approaches to guarantee high-quality and pure final extracts. Technologies such as supercritical fluid extraction (SFE), microwave-assisted extraction (MAE), and ultrasonic-assisted extraction (UAE) are noteworthy examples that are applicable to these matrices. The systematic flow of the anthocyanin extraction methods are presented in Figure 1. These methods are distinguished by their lower energy consumption, lower amounts of organic solvents used, and shorter operating times, all in line with sustainability principles (Benucci et al., 2022). Advancements in extraction technology have led to an increased interest in the nutritional and medical significance of extracted ANC. A critical comparison of the novel extraction techniques for anthocyanins is summarized in Table 1.

**Table 1:** Critical comparison of novel anthocyanin extraction techniques

**Extraction Techniques**	**Strengths**	**Weaknesses**	**Industrial Applicability, Cost-Effectiveness & Environmental Impact**	**References**
**SFE**	Employs CO₂ as a solvent, easily removed post-extraction and reduces deterioration due to heat. Extraction time can reach 1 hour. Does not require alternative energy sources and is scalable for larger operations.	To extract polar compounds, a co-solvent is necessary. The quantity and type of co-solvent must be optimized alongside other parameters. Subcritical water extraction (SWE) requires high temperatures to reach subcritical conditions, though ethanol may be used as an alternative.	Shows high **industrial scalability**, particularly for thermolabile compounds. However, the high **initial equipment cost** and need for co-solvents reduce cost-effectiveness. Environmentally, it is **favorable** due to CO₂ recyclability and low solvent residues.	(Muangrat et al., 2017)
**UAE**	Versatile, adaptable, low cost, ease of use, rapid energy transfer, minimal solvent use, short extraction time (5–60 min). Scalable for large-scale applications; enzyme-assisted UAE can enhance bioactivity extraction.	Lacks homogeneity unless probe systems (PUE) are used, which increases cost and complexity. Filtration and cleanup steps required; possible operator fatigue.	Considered **cost-efficient** and already applied in industry. However, **non-linear energy distribution** may limit uniform large-scale processing. Environmental impact is **moderate**, depending on solvent use and post-treatment steps.	(Kumar, Srivastav, and Sharanagat 2021; Decker et al., 2024; Benchikh et al., 2021)
**PLE**	Protects light- and oxygen-sensitive compounds; uses low solvent volumes. Scalable for industrial use with temperature control.	Requires expensive equipment and post-extraction cleanup (1–2 hours).	Suitable for **high-value products**, but **capital cost is high**, which limits adoption in low-cost food matrices. Solvent reduction offers **environmental benefits**, but extended cleanup steps reduce its process efficiency.	(Mehta et al., 2022)
**MAE**	Fast and uniform heating with minimal solvent and short extraction times (1–40 min). Vacuum microwave extraction allows lower temperatures and scalability for industrial use.	Solvents must be microwave-absorptive. Heating may damage compound structure/function. Post-extraction filtration and cleanup needed.	Demonstrates **good scalability**, especially with continuous-flow systems. Energy use is moderate, but thermal degradation risk limits industrial adoption. **Cost-effectiveness depends on reactor type**, while solvent selection affects environmental sustainability.	(Ferreira et al., 2020; Barroso et al., 2023; Zhang et al. 2025; Wang, Li, et al., 2024; Xue et al., 2021)
**HHPE**	Very short processing time (∼5 min) at room temperature; high repeatability and reduced solvent use. Suitable for large-scale applications.	High maintenance and equipment costs; high pressure may alter compound structure or function, requiring optimization.	High-pressure technologies are **already used in food preservation**, supporting industrial applicability. However, **operational costs are high**, and energy consumption may be significant. Solvent-free nature makes this method **environmentally favorable**.	(Tena & Asuero, 2022)
**PEFE**	Extremely fast extraction (<1 s) at room temperature; low energy use and reduced operational costs. Feasible for large-scale implementation.	High electric fields can damage compounds; matrix resistance must be reduced. Industrial use faces challenges (cooling systems, pulse uniformity, limited solvent choices).	Highly promising for **rapid processing at industrial scales**, but **infrastructure requirements and safety measures increase costs**. Environmental footprint is low due to minimal solvent use, but energy demand during pulsing must be monitored.	(Mehta et al., 2022; Yusoff et al., 2022)
**HVED**	Low temperature, short extraction time, minimal energy input, scalable for large-scale use.	High maintenance requirements; free radicals generated may reduce antioxidant activity.	Industrial usage still in early stages; **maintenance complexity reduces cost-effectiveness**. The need to prevent oxidative degradation increases process control demands. Despite minimal energy use, **environmental concerns arise due to reactive species formation**.	(Jha & Sit, 2022)
**EAE**	Environmentally friendly and selective; when combined with ultrasound, improves bioactivity and yield.	Enzymes are expensive; activity depends on pH, temperature, and substrate. Long extraction times (1–12 h) and cleanup required.	Highly **eco-friendly** with selective extraction, but **commercial scaling remains limited** due to enzyme cost and stability issues. Long extraction time reduces **industrial feasibility** unless immobilized enzymes or enzyme recycling technologies are used.	(Gligor et al., 2019; Kim et al., 2024)

### 2.1 Solvent extraction

Among the conventional methods, the Solvent Extraction Method (SEM) functions according to the comparable solubility principle, with the choice of organic solvents being crucial. Frequently utilized solvents for extracting ANC’s include ethanol, methanol, acidified water, and acidified ethanol. ANC’s can be extracted via SEM under specific conditions, including 1:15–1:30 a solid–liquid ratio, 34.7–52.03°C temperature range for extraction, and duration of 5 minutes to 4.2 hours. SEM offers conveniences, such as easy operation, minimal equipment requirements, and straightforward implementation. However, it also has significant drawbacks, such as drawn-out procedures, inefficient extraction, excessive solvent usage, and the need for high temperatures (Johnson et al., 2020b).

### 2.2 Novel technique

Several novel technologies have been used for the extraction of ANC’s as they involve reductions in the quantity of organic solvents utilized, decreased exposure to reducing agents, and a diminished requirement for purification and concentration steps. These refinements contribute to an overall enhancement in the extraction yield, selectivity, and/or kinetics compared to classical and traditional approaches. Different sophisticated extraction strategies have been established and are frequently used to overcome the drawbacks of conventional solvent extraction procedures to boost the ANC output. Ultrasound-assisted extraction (UAE) improves the transmission of mass procedures, disassembles cell structures, and increases the extraction rate. Microwave Assisted Extraction (MAE) uses microwave radiation to quickly heat samples, aid in cell wall breakdown, and enhance compound extraction. Supercritical Carbon Dioxide Extraction (SCDE) uses supercritical CO_2_ as a solvent, offering advantages such as selectivity, low toxicity, and minimal alteration of the chemical constituents. Ultrasound-Assisted Enzymatic Extraction (UAEE) combines ultrasound and enzymes to damage cell membranes and improve the release of intracellular components. Ultrasound-Assisted Deep Eutectic Solvent Extraction (UADESE) combines deep eutectic solvents with ultrasound to improve the extraction of bioactive compounds. Ultrasonic microwave-assisted extraction (UMAE) synergistically combines ultrasound and microwave energy for faster extraction. These advanced methods aim to overcome traditional technique drawbacks, providing improved efficiency, shorter extraction times, and enhanced selectivity for ANC’s and other bioactive compounds (Azman et al., 2020; Ferreira et al., 2020; Herrera-Ramirez et al., 2020; Thilavech et al., 2025).

#### 2.2.1 Supercritical fluid extraction (SFE)

SFE has become a prominent and feasible technique in recent years. The extraction process in this approach is based on variations in solubility and involves the introduction of a supercritical fluid that serves as the medium for extraction of compounds with targeted components. Two main processes are involved in this process: first, the required component is retrieved from the supercritical fluid; next, the liquid is quickly evacuated by altering the pressure and temperature. CO_2_ is commonly used as a solvent in SFE owing to its non-explosive nature, ready availability, and ease of removal from the final extract. It is non-toxic, does not cause significant chemical changes in bioactive compounds, and preserves biological characteristics. However, since CO_2_ is non-polar, SFE works more effectively in extracting pigments that are non-polar in nature, such as chlorophylls and carotenoids, compared to those that are polar in nature, such as ANC and betalains. To extract ANC’s, co-solvents such as ethanol or methanol are often used to improve extraction efficiency (Jiao, 2018; Muangrat et al., 2017). Supercritical carbon dioxide possesses properties that bridge the gap between gases and liquids, setting it apart from traditional solvents. It has unique physicochemical attributes, such as robust solubility, high mass transfer coefficient, non-toxicity, cost-effectiveness, and easy accessibility. Consequently, SCDE, as an innovative extraction method, offers numerous benefits, including high efficiency, environmental sustainability, safety, and minimal environmental impacts. Moreover, SCDE operates at low treatment temperatures, making it particularly suitable for extracting thermally sensitive compounds, such as ANC (Reinoso et al., 2025).

#### 2.2.2 Ultrasound-assisted extraction (UAE)

The desired chemicals are extracted using solvents and ultrasonic radiation from a variety of plant matrices in a process called ultrasound-assisted extraction (Hao et al., 2023). Mechanical waves with wavelengths higher than 20 kHz or above the 20 Hz–20 kHz hearing range in humans are referred to as ultrasound. Compression and rarefaction cycles are involved in these waves, which can travel across liquid, solid, or gaseous media and push or dislodge molecules around them. When rarefaction occurs at high intensities, the attractive forces of the molecules are outweighed by negative pressure, leading to their separation and the formation of bubbles produced by cavitation. Heat patches and unfavorable local conditions are produced as these bubbles enlarge during coalescence and subsequently explode during compression. The temperature within these hot spots can increase to approximately 5000 K, accompanied by a pressure increase of as much as 1000 atm. These conditions accelerate biochemical processes (De Barros et al., 2024; Kumar et al., 2021b).

With the goal of increasing extraction efficiency and cutting down on time, ultrasonic-assisted extraction makes use of cavitation effects and powerful shear stresses generated by ultrasound between 20 kHz and 50 MHz (Yuzuak et al., 2023). According to a study of research on UAE ANC’s conducted both domestically and abroad, ultrasonography has the ability to somewhat enhance natural ANC extraction when compared to conventional approaches (Belwal et al., 2019; Wang, Han, et al., 2020). Nevertheless, it is important to note that the mechanical and cavitation impacts of ultrasound throughout the UAE method may affect the structural integrity of ANC. Hence, careful regulation of ultrasonic parameters, such as ultrasonic power, temperature during extraction, solid-to-liquid ratio, and duration of extraction, is crucial for optimizing the benefits of ultrasonic extraction while minimizing the risk of structural damage to ANC (Carrera et al., 2021; Liu et al., 2022).

#### 2.2.3 Microwave-assisted extraction (MAE)

MAE employs microwave radiation from electromagnetic sources, which are absorbed by molecules and transformed into thermal energy. The disintegration of the cell walls of the plants caused by this intense microwave heating caused the cell structures to expand. MAE involves applying non-ionizing electromagnetic radiation in a focused or non-focused manner at wavelengths in the 300 MHz–300 GHz range (Benucci et al., 2022; Mirzazadeh et al., 2024). The MAE process typically includes three main steps: isolating solubilized components from the sample matrix, elevating the pressure and temperature, and permitting the solvent to enter the matrix of the sample. Compared with other extraction methods, MAE has better recovery rates and is capable of extracting bioactive chemicals more quickly (Arruda et al., 2021; Jara-Palacios et al., 2019; Tena & Asuero, 2022). Several parameters influence the efficiency of MAE, such as temperature, microwave power, frequency, duration of irradiation, extraction pressure, solid-liquid ratio, solvent nature and composition, and matrix particle dimensions. It is important to select the right solvent; the most frequently used polar solvents are those with large dielectric constants, such as ethanol and water. MAE technology enhances the extraction of ANC’s by accelerating mass transfer rates through cell disruption caused by internal warming. This leads to a greater release of ANC’s into the extraction medium (Barroso et al., 2023; Jha & Sit, 2022; Yuzuak et al., 2023).

As microwave-assisted extraction (MAE) is more efficient and improves recovery compared to conventional procedures, it has become more popular as a promising technique. The fundamental processes that enable microwave extraction include the relaxation of dipoles and ion conduction in dielectric compounds (Pham et al., 2022; Zhang, Aniya, et al., 2025). The target chemical substances become more soluble when the solvent is quickly heated by microwave radiation, which also reduces the extract viscosity. Furthermore, it alters the cell structure of plants, which reduces the obstruction in the transfer of mass and promotes ANC diffusion. This ultimately boosts the extraction efficiency of target compounds (Wang, Li, et al., 2024; Yiğit et al., 2022). MAE can potentially damage ANC structures owing to excessive vibrations and high local temperatures. Therefore, careful control of parameters such as extraction time, solid-to-liquid ratio, and microwave power ratio is crucial to achieve an optimal ANC yield while minimizing structural damage (Feng et al., 2019; Xue et al., 2021).

#### 2.2.4 Pulse electric field (PEF)

Electrical current is used in a non-thermal extraction technique known as pulsed electric field to deliver high-voltage pulses across plant matter sandwiched between electrodes in milliseconds. This technique induces an electric potential across the cell membranes, resulting in electroporation and hydrophilic pore formation. Through the creation of irreversible or reversible holes, electroporation increases the permeability of cells and facilitates the migration of target molecules across the membrane. Consequently, PEF treatment increases the yield, mass transfer, and extraction rates while jeopardizing the structural integrity (Kurtulbaş Şahin et al., 2021; Saini et al., 2019). The PEF extraction efficiency is dependent on several factors, such as the temperature, treatment duration, pulse count, and particular energy input, combined with the pH, conductivity, ionic strength, and electric field strength of the medium. PEF extraction offers advantages, such as chemical-free processing, reduced operational costs, and environmental sustainability. Because it operates without heat, PEF is suitable for extracting thermally sensitive compounds, such as ANC’s (Arruda et al., 2021; Jha & Sit, 2022). PEF extraction faces challenges such as higher costs compared to thermal methods, requirements for low electrical conductivity and absence of air bubbles in samples, and the risk of ANC degradation into chalcones and other pseudobases at excessively high voltages. To optimize PEF extraction and preserve ANC’s, it is crucial to use low or moderate intensity to avoid degradation and achieve the maximum extraction yield (Mehta et al., 2022; Tena & Asuero, 2022).

#### 2.2.5 Enzyme-assisted extraction (EAE)

EAE is an increasingly common and affordable alternative to conventional solvent-extraction techniques. This technique employs enzymes, which are highly specific catalysts, to facilitate processes in moderate environments with low temperatures and short times (Yusoff et al., 2022; Kim et al., 2024; Amulya and ul Islam 2023; Gligor et al., 2019). Enzymes function by disassembling the intricate structural elements of the cell walls and hydrolyzing their components, leading to increased cell permeability and discharge of intracellular elements into the solvent used for extraction. Compared to conventional methods, EAE offers several advantages, including lower energy utilization, faster extraction rates, increased yields, and easier recovery through reduced solvent use compared to conventional methods (Allcca-Alca et al., 2021; Das et al., 2021; Rifna et al., 2023). Despite its benefits, EAE presents challenges. The cost of enzymes can be prohibitive, limiting their use to high-value compounds. Additionally, enzymes do not completely disrupt the cell matrix and require additional steps for sample separation and purification. Moreover, enzymes are not recyclable or reusable; after many uses, they lose their catalytic activity. These factors contribute to the relatively high cost of EAE and its limited suitability for large-scale industrial applications (Alvarez-Rivera et al., 2020; Lefebvre et al., 2021).

#### 2.2.6 Pressurized liquid extraction (PLE)

Pressurized liquid extraction is an additional extraction method, alternatively referred to as pressurized fluid extraction (PFE), high-pressure solvent extraction (HSPE), or accelerated solvent extraction (ASE) (Jha & Sit, 2022). This technique uses immense pressure to maintain the liquid solvent at temperatures significantly higher than its boiling point. This process enhances the ability of a substance to dissolve, facilitate, soak the sample, and enable its penetration into the matrix (Saldaña et al., 2021). High pressure reduces both the viscosity and surface tension, facilitating solvent infiltration into the solid matrix and accelerating the rate of diffusion and mass transfer. Concurrently, high-pressure leakage occurs when air escapes from plant cell vacuoles, causing denaturation of proteins found in cell membranes and increased access to the desired substances during extraction (González-de-Peredo et al., 2021).

This extraction process ensures improved solvent infiltration into the cell membranes to promote bio-accessibility, with pressures beginning at 100 MPa and increasing to values beyond 1000 MPa. According to the literature, higher hydrostatic pressure results in an increased release of cellular components and higher extraction efficiency, albeit potentially reducing selectivity (Carrera et al., 2021). A key advantage of pressurized liquid extraction is its versatility in using various solvents, which enables the recovery of polar and non-polar substances. Environmentally friendly solvents include ethyl acetate, water, ethyl lactate, and ethanol (Ochoa et al., 2020). Other benefits include lower solvent volume requirements, faster extraction periods, and improved bioaccessibility of the sample's bioactive chemicals. Nevertheless, the price of energy associated with the generation of higher pressures is a notable drawback of this extraction technique (Mirzazadeh et al., 2024; Saini et al., 2019).

#### 2.2.7 Ohmic heating-assisted extraction (OHM)

Electroconductive heating, generally referred to as ohmic heating-assisted extraction, leverages a food material's natural resilience to electric current to transform electrical energy into heat, which in turn enhances the permeability of the membrane. Although not widely employed for ANC extraction, some studies have been conducted in this area (Cabas & Icier, 2021). Although the rapid heating process of OHM causes less thermal damage, findings from different studies indicate that OHM may lead to degradation similar to that of conventional heating methods. Consequently, additional research is required to ascertain the effectiveness of OHM in ANC extraction processes (Jha & Sit, 2022; Picot-Allain et al., 2021).

### 2.3 Combined extraction methods

Recent technological progress has accelerated the development of combined extraction systems, which integrate complementary physical and biochemical mechanisms to significantly enhance anthocyanin (ANC) recovery from fruit and vegetable by-products. Among these, Ultrasound-Assisted Enzyme Extraction (UAEE) and Ultrasound-Assisted Deep Eutectic Solvent Extraction (UADESE) represent the most widely investigated hybrid strategies due to their ability to improve yield, selectivity and pigment stability while promoting sustainable processing practices (Li et al., 2023; Wu et al., 2023).

#### 2.3.1 Ultrasound-Assisted Enzyme Extraction (UAEE)

UAEE combines cavitation forces generated by ultrasound with enzymatic hydrolysis of plant cell wall components, resulting in efficient cellular disruption and enhanced mass transfer. Enzymes such as cellulase and pectinase degrade structural polysaccharides, while ultrasound facilitates solvent penetration and liberation of bound anthocyanins (Picot-Allain et al., 2021; Reinoso et al., 2025). This dual mechanism helps to overcome diffusion barriers that limit conventional extraction, particularly in matrices with high fiber or lignin content.

Experimental evidence supports this synergistic effect. For instance, extraction of mulberry wine residues using UAEE with cellulase–pectinase (1:1 w/w) at 45°C yielded 53% higher anthocyanin recovery compared with UAE alone (Gautam, 2024). Likewise, Picot-Allain et al. (2021) reported a 1.7-fold improvement in ANC extraction from raspberry pomace under optimized UAEE conditions (400 W ultrasound, 2% enzyme). The mild operating temperature (40–50°C) also preserves pigment integrity, reducing thermal degradation and oxidation during processing (Zhao et al., 2023).

#### 2.3.2 Ultrasound-assisted deep eutectic solvent extraction (UADESE)

UADESE integrates DES-based solubilization with ultrasonic cavitation, creating an eco-efficient alternative to traditional organic solvents. Deep eutectic solvents (DESs) composed of choline chloride, glycerol, lactic acid or glucose exhibit adjustable polarity, hydrogen bonding capacity and biodegradability, enabling selective recovery of anthocyanins without toxic solvents (Jha & Sit, 2022; Zhou et al., 2024). Ultrasound enhances DES penetration into plant tissues, improving dissolution and desorption of pigments.

Jha and Sit (2022) demonstrated that UADESE extraction of black rice bran using choline chloride–lactic acid DES under ultrasound conditions (40 kHz, 55°C, 25 min) increased ANC yield by 61% compared with conventional solvent extraction. Similarly, Diaconeasa et al. (2022) reported 2.3-fold higher anthocyanin recovery from black carrot residues and 35% lower energy consumption compared to DES-only extraction. These findings highlight UADESE as a promising green extraction platform suitable for upscaling if solvent recyclability and process energy balance are optimized (Xu et al., 2022; Feng et al., 2025).

#### 2.3.3 Other emerging hybrid methods

Methods such as Ultrasound–Microwave-Assisted Extraction (UMAE) and Enzyme–Microwave-Assisted Extraction (EMAE) have also shown enhanced extraction efficiency. UMAE combines rapid microwave heating with cavitational disruption, accelerating pigment release from complex matrices. Diaconeasa et al. (2022) reported up to twofold improvements in ANC recovery using UMAE for Lonicera caerulea compared with UAE or MAE alone. However, excessive microwave power may degrade thermolabile pigments, necessitating precise control of exposure time and temperature (Feng et al., 2025).

## 3 Purification process

The extraction techniques utilized for ANC isolation from plant matter lack specificity, leading to a final extract that contains substantial amounts of other components such as sugars and organic acids. Therefore, eliminating interfering substances after extraction is essential, particularly in studies on ANC properties, where expensive standards often hinder their widespread use (Liu, Liu, Wu, Zheng and Zhang, 2021, Liu et al., 2021; Fernandez-Aulis et al., 2019). Several approaches have been developed for ANC purification, ranging from complex chromatographic methods to solid-phase extraction (SPE) (Ferreyra et al., 2020).

The various purification approaches for anthocyanins differ considerably in selectivity, operational cost, scalability, and matrix compatibility, especially when dealing with complex fruit and vegetable by-products. With increasing emphasis on eco-efficient recovery methods, it is essential to critically evaluate the performance and limitations of each purification technique to improve downstream processing workflows and ensure industrial feasibility (Castro-Muñoz et al., 2016; Palos-Hernández et al., 2024).

### 3.1 Anthocyanins precipitation

Divalent lead (Pb) has been used to separate ANC’s from aqueous solutions as one of the initially introduced methods for purifying ANC’s. But other substances with carboxyl or nucleophilic groups, like fatty acids, phenolics, amino acids, organic acids, tannins, as well as flavonoids, also precipitate in the presence of lead, thus this approach doesn't just target ANC’s (Guo, Fan and Wang, 2022, Guo et al., 2022; Zorzi et al., 2020). As a result, this approach is largely used as an initial purification step, particularly because dominant impurities such as sugars remain unprecipitated by this method (Guimarães et al., 2023). Because of the dual nature of ANC pigments as both acids and bases, their interaction with Pb^2+^ varies with pH, whereas neutral and alkaline conditions facilitate precipitation more effectively than acidic environments. Proper pH regulation is essential, because each type of ANC exhibits unique optimal pH levels for precipitation, which may cause some to remain dissolved in the solution. ANC and lead salts were dissolved in an alcoholic solution containing either hydrochloric acid (HCl) or sulfuric acid (H_2_SO_4_). During this process, lead salt precipitates out, allowing for its removal and resulting in an alcohol-based suspension rich in ANC (Ding et al., 2020; Saldaña et al., 2021).

Although precipitation is widely used as an economical initial purification step, it is inherently non-selective, often leading to the co-precipitation of proteins, phenolic acids, and organic acids, which reduces purity (Molina et al., 2023). Efficiency is also strongly influenced by pH, ionic strength, and anthocyanin structure, making scale-up challenging. Furthermore, the use of toxic metal salts such as lead acetate restricts its application in food or pharmaceutical production, and therefore, this method is now mainly employed as a pre-treatment before chromatographic purification (Fenger, 2020; Yang, Ye, et al., 2025).

### 3.2 Solid phase extraction

Solid-phase extraction (SPE) is used to separate dissolved substances based on their physical and chemical properties. Adsorption materials, such as Sephadex, C18, and Amberlite resins, are used in conventional SPE. These materials interact with compounds via hydroxyl bonds or hydrophobic reactions involving aromatic rings along with solvents with different polarities present (Enaru et al., 2021; Tao et al., 2020). Several studies have explored the use of six different absorbent resins: Amberlite IRC 80 mild acidic anion exchanger, Dowex 50WX8 powerful acidic cation exchanger, Amberlite IR 120 mild acidic cation exchanger, non-polar silica gel, and non-ionic acrylic ester resins with medium polar (Amberlite XAD7 and XAD4). The results showed that the best adsorption levels and extraction efficiency (approximately 93% recovery) were achieved by non-ionic acrylic ester resins, whereas non-polar silica gel exhibited no significant ANC adsorption (Qi et al., 2023).

This method is economical, straightforward to implement, and highly reproducible, which explains its broad applicability. Nevertheless, owing to the lack of selective interactions, certain contaminants may persist (Banach et al., 2020; Silva, Reboredo & Lidon, 2022). For instance, after using Amberlite resin to filter blueberry extract, researchers detected chlorogenic acid (CGA) residues along with additional flavonoids. In response, an alternative SPE system was developed, capitalizing on the variable charges of ANC’s at different levels of pH. This novel technique combines cation exchange properties with reversed phase interactions in the absorbent. ANC’s, in their flavylium cation form, are injected at pH 2 and react with the negatively charged absorbent, permitting the degradation of other substances using solvents of varied polarity if the pH levels are maintained. (Tan et al., 2021). An alkaline eluent changes the ANC’s to negatively charged quinoidal bases, allowing them to be recovered without binding to the resin. Compared with typical SPE methods, this approach showed improved purity and extraction of ANC, sorbent capacity, cost-effectiveness, straightforwardness, and productivity. A comparably effective strategy was described by (Speciale et al., 2014). The dihydroxy structure of ANC’s was used to create stable metal complexes with Fe^2+^, Fe^3+^, Cu^2+^, Mg^2+^, and Ca^2+^ in alkaline environments. These complexes break down under acidic conditions (Stote et al., 2020). Although this process provides effective purification at a low cost, the matrix composition must be carefully evaluated, as various ANC’s may react significantly differently according to variations in pH and metal decomplexations, possibly leading to alterations in the ANC profiles (Le et al., 2023; Stote et al., 2020).

SPE is considered one of the most reliable and scalable purification techniques for anthocyanins, with non-ionic resins such as Amberlite XAD-7 and XAD-16 achieving recovery rates above 90% in berry and purple rice extracts (Hunter, 2020). However, because SPE resins exhibit limited molecular selectivity, additional steps such as gradient elution or two-stage purification are often required to obtain analytical-grade anthocyanins (Kammerer et al., 2019). For industrial use, optimization of resin regeneration cycles and solvent recovery is essential to reduce operating costs and ensure environmental compliance (Molina et al., 2023).

### 3.3 Countercurrent chromatography (CCC)

This chromatography method isolates large amounts of sample material using two phases of immiscible liquid under mild conditions. It has proven useful for handling sample amounts of several hundred milligrams in one operation (Abdin et al., 2020; Xu et al., 2022). High-speed CCC (HSCCC) has shown considerable potential for the extraction of massive amounts of ANC from sources such as blackberries and elderberries, which are positioned in a Teflon coil and rotated to create zones of mixing and separation among each of the liquid phases. This guarantees that the sample flows uniformly throughout both phases (Zhao et al., 2020). One of CCC's main benefits of CCC is its capability to operate without high-cost columns and its use of comparatively low-cost solvents, such as n-butanol, acetonitrile, water, trifluoroacetic acid (TFA), and methyl tert-butyl ether (MTBE) (Zhao et al., 2020; Silva, Reboredo & Lidon, 2022). The economic benefits of CCC make it a favorable option for industrial-scale production. A widely used solvent system, consisting of n-butanol, water acidified with TFA, tert-butyl methyl ether, and acetonitrile in a 2:5:2:1 ratio, can be used to extract ANC’s from products such as red cabbage, wine, and blueberries (Silva, Reboredo & Lidon, 2022).

CCC and HSCCC offer notable advantages by avoiding solid stationary phases, thus minimizing sample loss and enabling continuous processing. These techniques have achieved >95% purity for anthocyanins extracted from red cabbage, blueberries, and chokeberries when using optimized biphasic solvent systems such as *n*-butanol–water–TFA (Liu et al., 2024; Nunes et al., 2022). Although initial equipment costs are relatively high, CCC delivers strong separation efficiency, reproducibility, and low solvent consumption, making it attractive for large-scale anthocyanin purification (Zeng et al., 2025).

## 4 Quantification of anthocyanins

The AOAC International method for quantifying ANC is a spectrophotometric technique that is particularly suitable for liquid products because of its simplicity and speed (Liu, Liu, Wu, Zheng and Zhang, 2021, Liu et al., 2021). ANC’s consist of anthocyanidin (aglycon) bound to various sugar moieties. The most prevalent ANCs in plants are pelargonidin, cyanidin, malvidin, petunidin, delphinidin, and peonidin. More than 600 ANC’s have been discovered based on the quantity of sugar molecules and the acylation group type. Beyond their role as natural pigments and in functional foods, the precise identification and characterization of ANC’s are crucial for understanding their various structures (Fernandez-Aulis et al., 2019).

### 4.1 Spectrophotometric determination: AOAC international method

The AOAC International pH differential method is the most widely applied spectrophotometric technique for determining total monomeric anthocyanins. It relies on the reversible transition of anthocyanins between the flavylium cation (red, pH 1.0) and the colorless hemiketal/chalcone forms (pH 4.5), with results typically expressed as cyanidin-3-O-glucoside equivalents (Reboredo & Lidon, 2022; Zorzi et al., 2020).

However, several limitations have been reported. The method lacks specificity, fails to differentiate between anthocyanin derivatives, and is vulnerable to interferences from other phenolics and polymeric pigments (Chaves et al., 2022; Guo, Fan and Wang, 2022, Guo et al., 2022). Moreover, light-scattering effects in dairy, cereal, and emulsified matrices hinder reliability in these systems, restricting its application primarily to clear solutions (Teng et al., 2020). Despite its drawbacks, it remains a practical preliminary quantification method when rapid assessment is prioritized over detailed compositional analysis (Liu, Liu, Wu, Zheng and Zhang, 2021, Liu et al., 2021).

### 4.2 Chromatographic and mass spectrometric approaches

High-performance liquid chromatography (HPLC) and ultra-performance liquid chromatography (UPLC) are widely regarded as the gold standard for qualitative and quantitative profiling of anthocyanins. HPLC–DAD enables reliable quantification of individual anthocyanins when analytical standards are available (Li, Zheng, et al., 2022). In addition, UPLC technology provides improved separation efficiency and reduced run times, facilitating accurate identification in complex food matrices. Coupling chromatography with mass spectrometry has significantly advanced anthocyanin analysis. Techniques such as LC–MS/MS, UPLC–QTOF–MS, and Orbitrap–MS allow structural elucidation of isomers, acylated derivatives, and degradation products based on fragmentation patterns (Pape et al., 2024; Bennett et al., 2021). Their integration with chemometric tools such as PCA and OPLS-DA has also enabled classification of anthocyanin profiles in relation to cultivar, geographical origin, and processing parameters (de Silva et al., 2022; Schnitker et al., 2024). These methodologies have therefore become essential for accurate quantification and in-depth characterization of anthocyanins in food systems.

### 4.3 Emerging analytical techniques

Advanced analytical platforms are increasingly focused on speed, sensitivity, and minimal sample preparation. Ion mobility spectrometry mass spectrometry (IMS–MS) introduces an additional gas-phase separation dimension that distinguishes anthocyanin isomers based on their collisional cross-sections (Schnitker et al., 2024). Ambient ionization techniques, including DESI–MS and DART–MS, enable direct surface analysis of foods without extraction, providing rapid in situ anthocyanin detection (Rasker et al., 2025).

Additionally, spectroscopic chemometrics coupling UV–Vis, Raman, and NIR spectroscopy with machine learning has emerged as a promising non-destructive method for quantifying anthocyanins in intact fruits, juices, and powders (Gong et al., 2025). These advancements offer new possibilities for rapid screening and real-time industrial monitoring of anthocyanin quality.

## 5 Anthocyanins stabilization

In acidic environments, ANCs predominantly exist as flavylium cations, which impart their characteristic deep red coloration. However, in alkaline aqueous solutions, these compounds undergo electron donation and are converted into quinonoidal bases, leading to a loss of color (Mattioli et al., 2020; Roy & Rhim, 2021; Yong & Liu, 2020). It is thought to be crucial to shield the cation flavylium from water and the nucleophilic attack of oxidants to preserve ANC (Cao et al., 2023). Several techniques have been devised to shield ANC’s in unfavorable environmental conditions, including: (1) chemical structure modifications through glycosylation and acylation (Luo et al., 2022); (2) the formation of stable complexes containing biological macromolecules, including polysaccharides and proteins (Dong et al., 2023; Koh, Xu, and Wicker 2020); (3) the application of micro- or nano-encapsulation techniques to ANC’s to form a physical protective shield that protects them from the stresses of their surroundings; and (4) co-pigmentation (Cao et al., 2023). These techniques increase ANC’s persistence and stability of ANCs throughout storage and processing, not only by reducing degradation but also by maintaining their structural integrity and functional qualities over time. Additionally, by protectinLuo ANC’s from the harsh gastrointestinal tract, encapsulation can increase their bioavailability and allow them to pass through the intestine to be absorbed by the blood. The shape, structure, and activity of the ANC-biopolymer complexes were identified by the parameters employed in the complexation process (Rocha et al., 2023).

### 5.1 Acylation

Acylated ANC’s display superior stability, biological activity, and bioavailability compared with their non-acylated forms. Numerous plant species naturally contain these compounds; however, they can also be synthesized using in vitro and in vivo techniques. There are four main ways to acylate ANC: (1) in vivo biosynthesis by genetically modifying plants to express acyltransferase genes, (2) semi biosynthesis, which mixes chemical synthesis and biosynthesis in vivo, (3) chemical-based acylation, and (4) enzyme-based acylation (Luo et al., 2022; Cai et al., 2022). For example, cyanidin-3-O-glucoside (raspberry ANC) was acylated using enzymes accompanied by methyl salicylate through a lipase-mediated reaction, achieving a conversion rate of (Teng et al., 2022). This acylation specifically targeted the C-6 position of the glucoside, leading to the formation of glucoside cyanidin-3-(6-salicyloyl). These compounds have shown enhanced stability in light, temperature, and oxidative environments, as well as enhanced oxygen radical absorbance capacity (ORAC) and greater ABTS and DPPH free radical scavenging activities (Dong et al., 2023; Teng et al., 2022).

Similarly, ANC’s in blueberries were acylated using maleic acid by a grafting solid-phase technique, which led to a higher degree of color stability in preservation compared to their unacylated counterparts (Fei et al., 2021). The grafted ANC’s using maleic acid preserved their color in the pH response, indicating their potential use in pH-sensitive color indicator packaging materials. Additionally, the enzyme-based acylation of ANC’s fractions in *Ribes nigrum* L. (blackcurrant), including delphinidin-3-O-rutinoside, cyanidin-3-O-rutinoside, cyanidin-3-O-glucoside, and delphinidin-3-O-glucoside with lauric acid, resulted in a marked improvement in lipid peroxidation inhibition and a substantial increase in thermostability (Yang et al., 2019). The effectiveness of anthocyanin (ANC) stabilization strategies largely depends on their underlying mechanisms at the molecular level. Acylation enhances hydrophobicity by attaching aromatic or aliphatic acyl groups to hydroxyl sites on the anthocyanidin skeleton, providing steric hindrance and hydrophobic shielding that limit hydration and nucleophilic attack on the flavylium cation (Luo et al., 2022). Recent enzyme-mediated acylation using lipases and acyltransferases has achieved regioselective modification under mild conditions, yielding acylated anthocyanins with improved colour retention, antioxidant activity, and gastrointestinal stability (Zhao et al., 2022).

### 5.2 Protein and polysaccharide binding approaches

Protein- and polysaccharide-binding methods are effective strategies to increase ANC’s nutritive qualities, protection, bio-accessibility, and bioavailability of ANC. Relationships between exogenous proteins and ANC, in particular, can improve the structural and functional attributes of these proteins, making them valuable for food production and development (Li et al., 2023; Ma et al., 2023; Jiao, 2018). The type of protein or polysaccharide polymer used in these complexes greatly influences the heat stability of the bound ANCs. For example, when blueberries were treated with xanthan gum instead of β-glucan and konjac glucomannan, ANC’s exhibited superior thermostability (Dong et al., 2023). Blueberry pectin was found in two different fractions: water-soluble fractions (WSF-P) and chelator-based fractions (CSF-P). CSF-P has a stronger binding capacity with ANC than with WSF-P, which provides better stability and protection against degradation under simulated gastrointestinal conditions (Koh, Xu, and Wicker 2020). Intermolecular interactions between ANC’s and proteins or polysaccharides are primarily non-covalent, involving electrostatic interactions, hydrogen bonds, and van der Waals forces (Dong et al., 2023). Under specific conditions, the formation of covalent connections between ANC and nucleophilic molecules in amino acid sequences can alter the function associated with ANC-protein complexes, which can affect their absorption, digestibility, and antioxidant properties (Ma et al., 2023). Covalently bound complexes may also become discolored owing to oxidation and quinone production. It is suggested that ANC-protein interactions are primarily non-covalent in acidic environments, while in alkaline conditions covalent linkages are more common ((Hoskin, Xiong, and Lila, 2019). In one study, a mixture of rice-pea protein isolated from muscadine grape pomaces and wild blueberries led to increased recovery, anti-inflammatory bioactivity, and antioxidant activity after digestion. After digestion, the recovery index for total phenolics for the percentage of blueberry protein-polyphenol particles and muscadine grape was 69% and 62%, respectively, whereas the unaltered pulpy residue extracts showed an index of 31% and 23%, respectively. Furthermore, compared with the digests of the unmodified extract, the digests of protein-polyphenol particles maintained a 1.5 to 2 times higher antioxidant capacity (Zhao et al., 2020). Protein and polysaccharide complexation relies primarily on non-covalent interactions (hydrogen bonding, hydrophobic stacking, and electrostatic forces) between anthocyanins and food macromolecules (Cheng et al., 2023; Ma et al., 2023). These complexes form protective microenvironments that reduce oxidation, aggregation, and pigment bleaching, while modulating release kinetics in the gastrointestinal tract. Recent findings indicate that β-lactoglobulin–anthocyanin nanocomplexes and xanthan gum–anthocyanin conjugates substantially improve colour stability, heat resistance, and antioxidant retention during simulated digestion (Solak et al., 2023; Xue et al., 2024). In addition, covalent conjugation through quinone-mediated coupling has emerged as a strategy to create highly stable anthocyanin–protein conjugates with controlled digestibility and improved antioxidant delivery (Wu et al., 2023).

## 6 Co-pigmentation

During co-pigmentation, ANC’s combine with other colorless pigments or organic acids and are involved in the formation of stable complexes through non-covalent interactions. The stability of ANC and color expression in plants, as well as different solutions, depend on this mechanism (Maciel et al., 2018). π-Conjugated systems found in co-pigments usually interact with ANC’s through π-π stacking, protecting them from nucleophilic attacks by water molecules, thus enhancing their stability (Cao et al., 2023). Common co-pigmentation methods include metal complexation, self-association, and intra- and intermolecular co-pigmentation (Cai et al., 2022). Amino acids, organic acids, polysaccharides, flavonoids, and phenolics are among the substances that can be used to create ANC copigments (Cao et al., 2023). Using van der Waals forces, hydrogen bonds, and electrostatic interactions, these co-pigments combine with ANC’s to form stable complexes. This interaction causes the maximum visible absorption wavelength (λmax) to shift towards longer wavelengths (bathochromic effect) and increases the pigment absorption (hyperchromic effect) across the visible light spectrum. In the wine industry, the darker hues and stronger purple tones that arise are significant (Maciel et al., 2018). The intermolecular communication co-pigmentation between malvidin-3-O-glucoside and (-)-epicatechin resulted in the greatest bathochromic shift of 13 nm and hyperchromic change of 142.46% when compared with the different ANC mono-glucosides and phenolics present in red wine. Similarly, a bathochromic shift of 6 nm was observed when chlorogenic acid was added to an extract rich in ANC from *Hibiscus sabdariffa* (Cao et al., 2023). Stability and color expression during co-pigmentation are mainly determined by the structure of pigments, particularly the B-ring hydroxylation and methoxylation patterns, along with the nature of the co-pigments (Saini et al., 2024). A study on the co-pigmentation of mulberry fruit juice ANC’s, primarily composed of cyanidin-3-glucoside, revealed that the addition of rutin, hyperoside, kaempferol, isoquercitrin, and quercetin leads to a stronger binding affinity and enhanced thermostability compared to catechin and quercitrin (Cao et al., 2023).

Co-pigmentation operates mainly through π–π stacking and hydrogen bonding between the anthocyanin chromophore and planar co-pigments such as flavonoids, phenolic acids, and certain amino acids. These interactions stabilise the flavylium cation, producing bathochromic (colour shift to longer wavelengths) and hyperchromic (increased intensity) effects that enhance colour stability under fluctuating pH and temperature (Bingöl et al., 2022; Gençdağ et al., 2022). Advances in computational and spectroscopic modelling have provided energy-minimised configurations of anthocyanin–phenolic complexes, enabling predictive design of co-pigments with enhanced binding affinity and tailored colour properties (Erşan et al., 2022).

## 7 Micro/nanoencapsulations

Nano/microencapsulation has demonstrated significant efficacy in mitigating the chemical instability of ANCs, a factor that hinders their bioavailability and integration in food systems. Various encapsulation techniques have been investigated, including emulsification, gelation, spray drying, freeze-drying, and complexation of polyelectrolyte (Sharif et al., 2020; Gençdağ et al., 2022; Milea et al., 2020). One of these approaches, spray-drying, has high encapsulation effectiveness; it is the most commonly used method for microencapsulating ANC’s. Common encapsulating agents include carbohydrates such as maltodextrin, gum Arabic, and maize, along with proteins like whey protein concentrate, gelatin, and soy protein isolate (Sharif, Khoshnoudi-Nia, and Jafari, 2020). The selection of the wall material in nano/microcapsules is critical as it dictates the physical properties of the capsules, thereby influencing the degradation kinetics of ANC’s. Microcapsules with elevated moisture content exhibit accelerated ANC degradation (Pieczykolan and Kurek, 2019). In a study focused on the microencapsulation of chokeberry using maltodextrin as the encapsulating agent, it was observed that after exposure to light and air for seven days, guar gum resulted in the lowest moisture content (1.66%), the greatest encapsulation performance (92.98%), the smallest particle size (16.29 μm), and the lowest disintegration rate (5.61%). However, throughout the same period, Arabic gum produced the largest amount of moisture (2.73%), weakest encapsulation effectiveness (78.61%), largest particle size (53.09 μm), and fastest disintegration rate (20.42%). To stabilize ANC’s in black raspberry, Shaddel et al. (2018)used a dual emulsion technique, a main water-in-oil (W/O) emulsion and an additional water-in-oil-in-water (W/O/W) emulsion Subsequently, they employed intricate coacervation using gum arabic and gelatine. The resultant ANC-containing microcapsules demonstrated increased storage stability, with an increase of up to 23.66%, after two months of storage at 37°C.

Emulsification, internal gelation, and spray drying were used to encapsulate the grape skin ANC extract in sodium alginate. This approach produced improved stability against light, heat, and pH stress, as well as increased encapsulation efficiency when compared to products produced by freeze-drying (Zhang, Celli, & Brooks, 2019). Furthermore, a maximum retention rate of 24.5% was observed for the freeze-dried powder throughout the last stage of gastrointestinal breakdown. When in vitro simulated gastric and intestinal digestion was performed, non-encapsulated and freeze-dried ANC showed retention efficiencies of only 15% and 1%, respectively.

Nanoparticles containing red raspberry pomace ANC extract and β-lactoglobulin (β-Lg) were synthesized via desolvation, followed by ultrasonication. These nanoparticles demonstrated superior stability in simulated oral (pH 6.8), intestinal tract (pH 6.9), and gastric (pH 2) environments, with a higher retention rate than unencapsulated ANC’s (Salah et al., 2020). Furthermore, the bioavailability of ANC-loaded β-Lg nanoparticles was markedly enhanced, with a bioavailability rate of 19.23%, as opposed to 11.27% for their unencapsulated counterparts.

Micro and nanoencapsulation provide a physical barrier against oxygen, light, and pH shifts. Techniques such as spray drying, liposomal encapsulation, ionic gelation, and nanoprecipitation have been successfully used to preserve anthocyanins during processing and storage by entrapping them within carbohydrate-, protein-, or lipid-based matrices (Jiang & Zhang, 2023; Rashwan et al., 2023). Emerging nano-delivery systems employing biopolymer–lipid hybrids or pH-responsive hydrogel nanoparticles show superior bioaccessibility, often achieving more than twofold higher in vitro absorption compared with non-encapsulated anthocyanins (Li et al., 2024; Salah et al., 2021). Encapsulation combined with biopolymer complexation, for example β-lactoglobulin or chitosan-coated nanoparticles, has further improved anthocyanin retention during gastrointestinal transit and enabled controlled release in the intestinal environment (Cheng et al., 2023; Tong et al., 2022).

## 8 Applications of anthocyanins

Naturally occurring ANC’s, predominantly sourced from blue, purple, or deep red fruits and vegetables, such as blackberries, red kale, blueberries, beetroot, black goji berries, purple potatoes, mulberries, and grapes (Halliwell, 2024), are widely regarded as safe food additives/colorants because of their antioxidant, anti-cancer, antibacterial, and metabolism-modulating properties.

However, the practical application of natural ANC’s is often constrained by their inherent instability and limited solubility in lipids. Consequently, there has been considerable research interest in acylated ANC’s, which have been recognized for their improved stability under various conditions. Commonly consumed acylated ANC’s typically comes from sources such as black carrots, red cabbages, radishes, purple sweet potatoes, and red potatoes (Zang et al., 2022).

### 8.1 Food colorants

Food color is a critical sensory characteristic that significantly influences consumer choices. Owing to safety concerns related to artificial coloring agents for food, such as lemon yellow, there has been an increasing shift towards the use of organic pigments, such as acylated derivatives of ANC’s, which are valued for their safety and health benefits (Xiong et al., 2019; Novais et al., 2022). Several studies have examined and documented the use of acylated ANC’s and their derivatives as naturally occurring colorants in food (Luzardo-Ocampo et al., 2021; Kumar et al., 2023; Vidana Gamage, Lim and Choo 2022). Two examples of acylated ANC’s are peonidin-3-(6"-hydroxybenzoyl)-sophoroside-5-glucoside and peonidin-3-(6"-hydroxybenzoyl-6"-caffeoyl)-sophoroside-5-glucoside that Oliveira et al. (2019) identified from sweet potatoes with purple flesh. These acylated compounds exhibited greater resistance to pH fluctuations than their non-acylated counterparts, thereby effectively preserving the blue and red colors in both basic and acidic conditions. Similar results were reported by Mendoza et al. (2018), who observed that the durability of the purple and blue colors in heavenly blue ANC’s was mostly attributable to the third position of their peonidin chromophore, which included three acylated sugar units. These sugar units facilitated the formation of protective intramolecular sandwich-type stacking around the flavylium cations, thus enhancing the color stability of the acylated ANC’s. This discovery may enable the more effective utilization and modification of various paeoniflorin sources for the development of stable and effective food colorants.

Additionally, Quan et al. (2019) studied ANC fractions isolated from the flesh of purple sweet-potatoes and evaluated how well they stored for six months at three different temperatures: 25°C (placed in darkness), 37°C (placed in darkness), and 25°C (placed in artificial illumination). Acrylated ANC’s, especially those containing derivatives of disaccharide and diacylated paeoniflorin, were shown to exhibit superior stability with significantly lower degradation rates after six months of storage compared to other ANC’s. Similarly, Venter et al. (2022) demonstrated that acylated ANC’s extracted from plants belonging to the *Lamiaceae* and *Geraniaceae* families maintained stability over an extensive range of temperatures, indicating the potential of these plant families as sources of organic food colorants.

Furthermore, several natural food-coloring agents have been used in the patents. A patent for an ANC-based colorant comprising pelargonidin-based and acylated ANC’s was obtained by Sendri and Bhandari (2024). This colorant has a characteristic red color that may be used in drinks, fruit plants, dairy goods, desserts, and confections. Black goji berries were the source of another patent for an organic pigment that preserved the stability of the color for over 14 days (Chai, Gong, Qian, Cao and Qian, 2021, Chaves, Monteiro and Oliveira, 2022). These patents were successfully utilized for several culinary items covering both mixes. including non-acylated ANC’s and natural food colorants rich in acylated ANC’s. These provide important information for the creation of organic colorants. The evaluated literature offers a summary of the industry's current situation, as well as future directions for commercial organic ANC colorants, with a focus on enhancing color diversity, stability in food matrices, and overall consumer acceptance.

### 8.2 Functionalizing agents

Epidemiological studies have established a correlation between ANC consumption and a decreased incidence of regressive and long-term illnesses, underscoring the potential of Ac-ANC as a functional agent. These compounds exhibit potent antioxidant properties, which suggest their use in reducing the risk of heart attack, stroke, neurological disorders, insulin resistance, diabetes, inflammation, and tumors (Tan et al., 2021; Shi, chen and chen 2021; Mattioli et al., 2020). During metabolic processes, cells generate various free radicals responsible for biomolecular oxidation, including proteins, lipids, carbohydrates, RNA, and DNA. Oxidative stress is implicated in Alzheimer's disease, cancer pathogenesis, autoimmune disorders, diabetes, and other conditions (Li et al., 2019). The ingestion of ANC’s, including their acylated derivatives, has demonstrated the ability to reduce the possibility of chronic illnesses owing to their robust antioxidant activity and efficacy in scavenging free radicals.

The molecular structure and purity of ANC’s are directly correlated with their antioxidant potential. Generally, a simpler molecular structure and higher purity are correlated with enhanced antioxidant activity (Oancea, 2021). Furthermore, ANC aglycones demonstrate superior antioxidant activity compared to their glycoside counterparts (Deepa et al., 2023) monoglycosides exhibit greater antioxidant potency than polyglycosides (Shi, chen and chen 2021) and Non-acylated ANC’s are more effective antioxidants than their acylated forms (Deepa et al., 2023) This efficacy is largely attributable to hydroxyl groups found in phenol within the structure of ANC, which are crucial in the ROS scavenging mechanism. In particular, the 3',4'-catechol configuration on ring B of ANC can create stably connected semiquinone or orthoquinone complexes that minimize intramolecular energy and promote an equitable distribution of the electron cloud through two successive single-electron transfer processes. The phenol hydroxyl group on ring A at the fifth position is extremely oxidation-susceptible, producing hydrogen ions and exhibiting a significant ROS-capture ability. Furthermore, the phenol hydroxyl groups at positions 3, 5, and 7 react with ROS to generate pseudo-semiquinone compounds. Keto-enol tautomerization increases the stability of these structures (Mattioli et al., 2020). Every ortho-substituted diphenol group is capable of scavenging up to four moles of ROS (Oancea, 2021). Temperature, light exposure, pH, and general storage conditions are some of the variables that affect ANC’s antioxidant activity of ANC during storage (Mattioli et al., 2020; Yan, Zhong, et al., 2020). Interestingly, the physical state of ANC, whether in solid or liquid form, has a major impact on how effective they are as antioxidants in different pH situations. For example, liquid ANC extracts maintain their antioxidant activity more effectively than solid extracts stored in acidic environments (pH 1, 2, and 3) (Li et al., 2021).

The therapeutic potential of ANC’s has been explored in several studies, particularly in animal models. Hyperuricemia, a common condition arising from purine metabolism disorders, causes uric acid crystals to build up around and within the joints, which causes gout. Although allopurinol is widely used to treat hyperuricemia, it has been associated with negative outcomes in some individuals (Johnson et al., 2020a; Li et al., 2021; Chen, Xiang, Zhao, Liu and Jia, 2025, Chen, Song, Zhao, Qi and Wang, 2025; Liu, Liu, Wu, Zheng and Zhang, 2021, Liu et al., 2021; Ngamsamer et al., 2022). Notably, some reports suggest that allopurinol treatment can trigger severe side effects associated with drugs in certain individuals (Chen, Song, et al., 2025; Liu, Liu, et al., 2021), prompting the search for alternative therapeutic options.

Zhang, Wu, et al. (2019) examined the potential medicinal benefits of purple sweet potato-derived acylated ANC’s combined with allopurinol in the treatment of hyperuricemia and kidney inflammation in rats with hyperuricemia. Compared to allopurinol alone, the combination treatment was found to be more successful in lowering blood uric acid levels and minimizing kidney impairment, indicating a potential complementary treatment strategy for hyperuricemia. This suggests that ANC-rich foods could be developed into functional health products.

In addition to their role in managing hyperuricemia, significant promise for trauma healing has been demonstrated by ANC’s, as demonstrated in animal studies. According to Tsutsumi et al. (2019), when rats were fed purple carrots orally, the acylated ANC’s in the carrots increased the circumferential blood flow in the cremasteric vein. This suggests that adding these chemicals to health foods may hasten wound healing after surgery. Furthermore, the ingestion of acylated ANC-rich natural foods, including purple potatoes, has been associated with a reduction in the risk of obesity, reduced insulinemia, lower gastrointestinal blood glucose levels, and gastrointestinal inflammation (Liu, Liu, Wu, Zheng and Zhang, 2021, Liu et al., 2021). Considering these encouraging results from animal studies, more investigations should focus on human clinical trials and the possible incorporation of acylated ANC’s into medications or medical nutrition regimens.

### 8.3 Nutraceutical potential of anthocyanins

Regular consumption of foods with high ANC has been associated with better lipid metabolism, enhanced vascular function, and pronounced antioxidant and anti-inflammatory effects, underscoring their potential application as nutraceuticals. The extensive literature highlights the historical utilization of ANC’s as phytopharmaceutical agents, appetite stimulants, and choleretic compounds, as well as their role in managing various pathological conditions. Studies conducted by (Sadowska and Bartosz 2024) and (Lakshmikanthan et al., 2024) demonstrated that ANC’s derived from vegetables, flowers, and blue-pigmented fruits exhibit a broad spectrum of health-promoting properties. These include antidiabetic, antimicrobial, anti-cancer, anti-inflammatory, and anti-obesity effects in addition to their potential to reduce the risk of cardiovascular diseases. These findings further supported their classification as nutraceuticals.

Similarly, research by (Mozos et al., 2021) established a correlation between ANC and flavanone consumption, and a reduced incidence of stroke and coronary artery disease in medical personnel. A total of 43,880 healthy males without a history of cancer or cardiovascular disease participated in the trial, and validated food frequency questionnaires were used to measure flavonoid intake. Additionally, a growing body of research indicates that ANC intake is positively correlated with a lower risk of non-fatal infarction of the heart, which is important for clinical trials aimed at treating acute coronary syndromes (Kim et al., 2021).

### 8.4 Active food packaging

The increasing incorporation of advanced and intelligent packaging systems within the food sector has led to the emergence of active food packaging as a viable approach for improving food preservation. This method incorporates functional components into packaging materials to release or absorb particular substances within the food matrix or its surrounding environment, thus prolonging shelf life and preserving product quality (Wu et al., 2021). Among the various bioactive compounds utilized in active food packaging, ANCs have attracted considerable interest because of their many uses. Zhai et al. (2017) investigated the application of intelligent food packaging by developing colorimetric films composed of polyvinyl alcohol and starch incorporated with roselle-derived ANC’s to monitor the quality of fish. According to this study, ANC-based films successfully decreased the amount of water and elongation of the film at the breakpoint. Over time, these colorimetric films exhibited notable color changes, attributed to interactions with the basic volatile amines present in the packaging environment.

ANC’s have been extensively documented for their potent antioxidant and antimicrobial activities, which further enhances their applicability to edible films. Sani et al. (2021) provided an example of this when they assessed the effectiveness of films made of carbohydrates and enhanced with red barberry ANC’s for use in food packaging. Their findings indicated that these films exhibit strong antioxidant and antimicrobial properties. Similarly, another study investigated the impact of cranberry-derived ANC’s on films of carboxymethyl chitosan (CMC) and chitosan hydrochloride (CHC) (Zhang et al., 2024). According to their research, the antioxidant capacity, thermal stability, and mechanical strength of CHC/CMC composite films were markedly improved by the addition of ANC. Furthermore, the inclusion of ANC-loaded CHC/CMC and gelatin-based films effectively mitigated oxidative degradation in stored olive oil, demonstrating that, during 56 days of storage, the peroxide value was 21.2 meq O₂/kg (Wang et al., 2019; Yong et al., 2022).

### 8.5 Bioavailability and bioaccessibility of anthocyanin

Anthocyanins (ACNs) exhibit substantial biological activity; however, their effectiveness in vivo is restricted by inherently low bioavailability and poor gastrointestinal stability. While the acidic gastric environment temporarily stabilizes the flavylium cation, rapid structural transformations occur in the small intestine due to neutral pH, digestive enzymes, and bile salts, leading to hydration, chalcone formation, and extensive molecular degradation (Kumkum et al., 2024; Li et al., 2023). Only a small fraction of intact anthocyanins is absorbed through transporter-mediated uptake, including bilitranslocase and sodium-dependent glucose co-transport pathways, whereas the majority reaches the colon unabsorbed (Liang et al., 2024). In the colon, bacterial glycosidases produced by genera such as *Lactobacillus*, *Bifidobacterium*, and *Akkermansia* transform ACNs into smaller, more stable phenolic metabolites—such as protocatechuic, syringic, and vanillic acids—which exhibit enhanced antioxidant, anti-inflammatory, and neuroprotective effects compared with their parent compounds (Godos et al., 2025; Chen, Xiang, Zhao, Liu and Jia, 2025, Chen, Song, Zhao, Qi and Wang, 2025).

To address low stability and improve intestinal retention, recent research has explored advanced delivery systems capable of protecting ACNs throughout digestion. Nanoliposomes, nanoemulsions, and protein–polysaccharide nanocarriers have been shown to markedly enhance physicochemical stability, protect ACNs against thermal and oxidative degradation, and increase post-digestion release efficiency (Rahim et al., 2025; Zhang, Wang, et al., 2025). Double-layer nanoliposomes, modified with plant-derived biopolymers such as synanthrin or pea protein isolate, significantly improve encapsulation efficiency and storage stability of ACNs (Zhang, Zheng, et al., 2025). Similarly, gelatin–pectin hydrogels act as pH-responsive carriers that restrict pigment degradation in the stomach while ensuring controlled release in the intestine, supporting microbial conversion into health-promoting metabolites (Li et al., 2025). Cyclodextrin complexes and starch-based carriers further enhance solubility, thermal resistance, and oxidative stability of ACNs, making them highly effective for gastrointestinal delivery (Ali et al., 2024; Wang et al., 2025). Collectively, these strategies significantly improve ACN bioaccessibility, bioavailability, and bioefficacy, reinforcing the importance of encapsulation systems for maximizing their functional role in food and nutraceutical applications (Mohammadi et al., 2024).

### 8.6 Health benefits of anthocyanins on gut and intestinal health

ANC demonstrated numerous beneficial health effects owing to different biological mechanisms. They reduce the incidence of many diseases, such as diabetes mellitus (Tena & Asuero, 2022) malignant tumors (Bars-Cortina et al., 2022; Les et al., 2021) cardiovascular diseases (de Arruda et al., 2022; Mozos et al., 2021) neurodegenerative disorders (Fallah et al., 2020; Zhang, Wu, et al., 2019) and obesity (Salehi et al., 2020). These positive health effects are mostly related to their powerful antioxidant activities (Gao et al., 2025; Garcia & Blesso, 2021) and capacity to modify the gut flora (Speer et al., 2020).

ANC’s, a class of bioactive flavonoids, have attracted considerable interest because of their ability to modulate the gut and intestinal health of animals. Upon ingestion, dietary ANC’s undergo a complex metabolic process that begins with their intact form being absorbed by stomach mucosa. Enzymes hydrolyze them into phenolic aglycones within the small intestinal tract, specifically the jejunum, where they are subsequently absorbed (Hair et al., 2021; Tian et al., 2019). After entering the colon, unabsorbed ANC’s are broken down by the gut flora. β-Glucosidase enzymes found in certain bacterial genera, such as Bifidobacterium and Lactobacillus, help break down ANC into smaller compounds. This microbial metabolism modifies the composition of gut microbes by increasing the bioavailability of ANC’s and encouraging the proliferation of beneficial bacteria (Liang et al., 2024; Verediano et al., 2021; Ma et al., 2024).

Recent studies have demonstrated that ANC’s exhibit prebiotic activity, thereby supporting overall health. A review of 34 studies on the prebiotic effect of ANC found that ANC enhanced gut bacteria numbers, such as *Lactobacillus* and *Bifidobacterium* spp., suppressed dangerous bacteria, such as *Salmonella* spp. and *E. coli*, and improved the intestinal environment. ANC also increases the formation of short-chain fatty acids (SCFAs), which are represented by acetic, propionic, butyric, and lactic acids (Wang et al., 2022). This aligns with findings from other dietary interventions, such as ω-3 polyunsaturated fatty acids, which similarly modulate gut microbiome composition and metabolic output (Yang, Chen, et al., 2025).

In addition to *Lactobacillus* and *Bifidobacterium*, anthocyanins selectively modulate several other key bacterial taxa associated with gut homeostasis. Recent studies demonstrate significant increases in *Akkermansia muciniphila*, *Faecalibacterium prausnitzii*, and *Roseburia* spp., all of which are butyrate-producing organisms strongly linked with anti-inflammatory intestinal environments (Liang et al., 2024; Chen, Xiang, Zhao, Liu and Jia, 2025, Chen, Song, Zhao, Qi and Wang, 2025). Conversely, ACN-rich interventions consistently reduce the abundance of pathogenic or opportunistic species such as *Escherichia coli*, *Clostridium perfringens*, and *Desulfovibrio* spp. microbes associated with endotoxin production and epithelial barrier disruption. These shifts indicate that anthocyanins function not only as prebiotic substrates but also as modulators of microbial ecology, promoting a shift toward a more beneficial, metabolically active microbial community.

After ingestion, limited small-intestinal absorption leaves substantial anthocyanins to reach the colon, where bacterial β-glucosidases/esterases deglycosylate them to phenolic acids (e.g., protocatechuic, vanillic, syringic). These metabolites, together with intact pigments, reshape the microbiota (↑ *Bifidobacterium*, *Lactobacillus*, *Akkermansia*; ↑ SCFA producers *Faecalibacterium*, *Roseburia*; ↓ *Enterobacteriaceae/E. coli*), increasing SCFAs (acetate, propionate, butyrate), lowering luminal pH, strengthening tight junctions (ZO-1, occludin), and down-modulating NF-κB/IL-6/TNF-α ultimately improving gut homeostasis and systemic metabolic/immune tone (Liang et al., 2024).

ANC’s and their metabolites also display significant neuroprotective activity. For example, in Alzheimer's disease models, protocatechuic acid, a by-product of cyanidin 3-glucoside formed by intestinal microbes, has been shown to have neuroprotective effects. Multi-omics approaches in APP/PS1 mouse models have further elucidated the intricate interactions between gut microbiota shifts and metabolic alterations in neurodegenerative contexts (Cheng et al., 2022). In one study, mice bearing the APP/PS1 mutation treated with blueberry extract (150 mg/kg administered every 24 h over a 16-week period) showed lower p62 levels, indicating improved autophagosome breakdown and reduced neuronal injury. Likewise, protocatechuic acid reduced Aβ25-35 cytotoxicity in hippocampal neurons by boosting autophagosome breakdown and decreasing ROS and LDH levels (Gościniak et al., 2024).

The interaction between ANC and gut microbiota leads to the production of short-chain fatty acids (SCFAs) such as butyrate, acetate, and propionate through bacterial fermentation. By reducing luminal pH, these SCFAs are essential for preserving gut health, inhibiting the growth of harmful bacteria, and serving as an energy source for colonocytes, thereby strengthening the intestinal epithelial barrier (Verediano et al., 2021). This barrier function is crucial for preventing the translocation of pathogens and antigens that can trigger systemic inflammation. Furthermore, the metabolites derived from ANC metabolism exhibit anti-inflammatory and antioxidant properties, contributing to the overall improvement in gut health. Studies have demonstrated that dietary supplementation with ANC enhances the quantity of microorganisms that produce SCFA, such as Faecalibacterium prausnitzii, while reducing the prevalence of pathogenic species, such as Escherichia coli (Kapoor et al., 2023). Li, Wang, et al. (2022) demonstrated that extracts from black rice high in ANC considerably increased the number of Lactobacillus and Bifidobacterium in mouse guts, accompanied by elevated SCFA levels and improved intestinal integrity. Similarly, another study reported that blueberry ANC’s enhanced gut microbial diversity and reduced inflammation in a porcine model. These results highlight the potential benefits of ANC as a prebiotic-like compound that can modulate gut microbiota and improve intestinal health in animals, offering a promising avenue for dietary interventions in veterinary medicine (Liang et al., 2024).

The health effects of anthocyanins are largely mediated by their microbial catabolites rather than the parent compounds. During colonic fermentation, bacterial β-glucosidases convert ACNs into smaller phenolic acids such as protocatechuic, syringic, gallic, and vanillic acids, which exhibit superior stability across gastrointestinal conditions (Godos et al., 2025). These metabolites exert potent antioxidant, anti-inflammatory, and neuroprotective activities and are capable of crossing the blood brain barrier more efficiently than intact anthocyanins. This enhanced bioactivity explains why ACN-rich foods often yield stronger physiological effects than predicted from their low native bioavailability (Kumkum et al., 2024). These metabolites also serve as signaling molecules that regulate microbial composition and intestinal immune responses, further reinforcing their relevance in gut health modulation.

ANC’s, a class of bioactive flavonoids, have demonstrated significant potential in enhancing animal health through their multifaceted effects on the gut microbiota, immune function, and systemic physiology. Studies have repeatedly demonstrated how ANC’s can alter the composition of the gut microbiota by encouraging the growth of good bacteria, such as *Lactobacillus* and *Bifidobacterium*, while suppressing harmful species, such as *Clostridium perfringens* and *Escherichia coli* (Jayarathne et al., 2019; Zhang, Chang, et al., 2022). This microbial modulation is facilitated by the enzymatic activity of gut bacteria, particularly β-glucosidase, which converts ANC into bioactive metabolites. In addition to the increased synthesis of short-chain fatty acids (SCFAs), such as acetate, butyrate, and propionate, these metabolites are essential for preserving intestinal health. SCFAs lower luminal pH, inhibit the proliferation of pathogenic bacteria, and serve as an energy source for colonocytes, thereby strengthening the intestinal epithelial barrier (Chen et al., 2024; Nathan et al., 2024). Similar barrier-strengthening and immunomodulatory effects via microbiota modulation have been reported with other bioactive interventions, in the context of type 2 diabetes (Su et al., 2023).

In addition to their prebiotic-like effects, ANC’s exhibit strong anti-inflammatory and antioxidant properties. Research has indicated that ANC inhibits oxidative stress indicators, such as malondialdehyde, while increasing the activity of antioxidant enzymes, such as glutathione peroxidase and superoxide dismutase (Tian & Lu, 2022). Furthermore, ANC suppresses the expression of pro-inflammatory cytokines, including TNF-α and IL-6, thereby mitigating inflammation under conditions such as colitis and heat stress. This anti-inflammatory action may involve modulation of key signaling pathways such as TLR4-NF-κB, which is known to be dysregulated in inflammatory bowel conditions through mechanisms like N4BP3-mediated IκBα ubiquitination (Jiang et al., 2025). These effects are particularly beneficial in improving gut barrier function, as evidenced by increased villus height and enhanced expression of occludin and zonula occludens-1, two tight junction proteins (Meineri et al., 2022). Beyond gut health, ANC’s have been shown to enhance immune responses by increasing immunoglobulins (IgA and IgG) and activating immune-related genes, as has been observed in swine and fish models. In companion animals, ANC’s have demonstrated anti-cancer properties, inhibiting tumor growth, improving gut microbiota composition, and reducing systemic inflammation. The gut-muscle axis represents another systemic pathway through which microbiota modulation may influence physiological functions, including muscle performance and meat quality (He et al., 2019; Liu et al., 2023) Collectively, these findings underscore the potential of ANC as a natural dietary intervention to improve gut health, immune function, and overall physiological well-being in animals, offering a promising avenue for application in veterinary medicine and animal nutrition (Csernus & Czeglédi, 2022; Fang et al., 2023).

Acylated ANC’s might have a greater effect on metabolic activity, inflammatory processes, and intestinal microbiota in people with diabetes mellitus type 2 in comparison with non-acylated ANC’s (Li, Wang, et al., 2022). For instance, polyphenols, such as ANC, influence the quantity of beneficial intestinal bacteria to produce prebiotic effects. ANC’s in blueberries are rich in malvidin-galactoside and malvidin-glucoside, and have been found to increase the levels of *Bifidobacterium* spp., helping to restore gut balance (Chen et al., 2023).

The extract of black currant, which is rich in ANC, inhibits α-glucosidase activity, considerably reducing postprandial hyperglycemia. Green currants with lower ANC levels have fewer benefits. Both green and black currants contain polyphenols, which may influence glucose transporters and salivary α-amylase (Krikorian et al., 2020)

A common complication of diabetes mellitus type 2 is a condition called diabetic retinopathy, which leads to vision impairment and is driven by elevated blood glucose levels (Do Rosario et al., 2021). Extracts from blueberries containing malvidin 3-glucoside, malvidin 3-galactoside, and malvidin ANC have been shown to reduce glucose-induced cytotoxicity and lower the expression of NF-κB, ICAM-1, and Nox4, and the levels of ROS and NO in human capillary endothelial cells in the retina via their anti-inflammatory and antioxidant actions (Krikorian et al., 2020).

Neurodegenerative illnesses, such as Alzheimer's disease, are linked to glycation, a process whereby sugars attach themselves to proteins, lipids, or nucleic acids. Glycation intensifies the deleterious consequences of proteins, such as β-amyloid (Aβ). Research on berry extracts has revealed that ANC’s are more effective than ANC-free extracts in trapping reactive carbonyl species, scavenging free radicals, and inhibiting Aβ fibrillation. ANC inhibited the generation of ROS produced by H2O2 and nitric oxide species caused by LPS at a dose of 100 μg/mL. In BV-2 microglia, 20 μg/mL ANC reduced caspase-3/7 activation and H2O2 cytotoxicity (Jati, 2022).

## 9 Research gaps and future perspectives

Despite substantial progress in anthocyanin extraction, stabilization, and characterization, several important gaps remain. From a process-engineering standpoint, most studies still rely on empirical, one-factor-at-a-time or response surface methodology (RSM) optimization, whereas recent work clearly shows that machine learning and hybrid AI models can more efficiently optimize multi-parameter extraction systems, including ultrasound-, microwave- and DES-based processes (Ayres et al., 2024; Korkmaz et al., 2025; Mantiniotou et al., 2024; Wang, Cui, et al., 2024). Future research should integrate AI-driven, multi-objective optimization frameworks that simultaneously consider yield, co-extracted impurities, energy use, and solvent recyclability, particularly for complex fruit and vegetable by-products. On the biological side, although meta-analyses and clinical trials suggest that anthocyanin supplementation can beneficially modulate lipid profiles, vascular function, and glycaemic control (Borda et al., 2025; Laudani et al., 2023; Mao et al., 2023; Neyestani et al., 2023), long-term, dose-response trials using well-characterized, matrix-bound and encapsulated anthocyanins are still scarce. Such studies are needed to link specific anthocyanin structures, metabolites, and delivery systems to clinically meaningful endpoints across diverse populations. In parallel, the translation of waste-derived anthocyanin ingredients into commercial nutraceuticals and functional foods faces regulatory and safety hurdles, including demonstration of contaminant control, batch-to-batch consistency, and toxicological safety of novel extraction solvents and carriers (Vilas-Boas et al., 2021; Messinese et al., 2024; Faraoni et al., 2024).

## 10 Conclusion

Anthocyanins (ANCs) are valuable natural pigments with demonstrated antioxidant, anti-inflammatory, and therapeutic effects, making them strong candidates for functional foods and nutraceuticals. However, their poor stability, low bioavailability, and sensitivity to environmental and gastrointestinal conditions limit their broader application in food and pharmaceutical industries. Although emerging extraction technologies such as ultrasound, microwave, enzyme-assisted, and pulsed electric field methods offer improved sustainability, they often fail to yield significantly higher efficiency than optimized conventional approaches. Current research remains largely laboratory-based, with insufficient emphasis on scalability, clinical validation, regulatory compliance, and standardized analytical protocols. Therefore, future studies should prioritize multi-level stabilization strategies, AI-driven extraction optimization, nanoencapsulation and acylation techniques, and long-term clinical trials, while integrating techno-economic and regulatory assessments. Addressing these gaps will be critical for translating anthocyanins into commercially viable, health-promoting ingredients for next-generation functional food systems.

## CRediT authorship contribution statement

**Hudda Ayub:** Writing – review & editing, Writing – original draft, Resources, Investigation, Formal analysis, Data curation. **Husnat Ahmad:** Writing – review & editing, Writing – original draft, Software, Methodology, Formal analysis, Conceptualization. **Syeda Hijab Zehra:** Writing – review & editing, Software, Methodology, Formal analysis, Data curation, Conceptualization. **Khadija Ramzan:** Writing – review & editing, Software, Investigation, Formal analysis, Data curation, Conceptualization. **Muhammad Adnan Arif:** Writing – review & editing, Validation, Resources, Methodology, Formal analysis, Data curation, Conceptualization. **Naima Tariq:** Writing – review & editing, Writing – original draft, Software, Methodology, Funding acquisition, Data curation, Conceptualization. **Maria Teresa Capucchio:** Writing – review & editing, Software, Formal analysis, Data curation, Conceptualization. **Robert Mugabi:** Writing – review & editing, Software, Methodology, Formal analysis, Data curation, Conceptualization. **Aanchal Sharma:** Writing – review & editing, Supervision, Resources, Investigation, Funding acquisition. **Gulzar Ahmad Nayik:** Writing – review & editing, Software, Formal analysis, Data curation, Conceptualization.

## Ethical approval

Ethics approval was not required for this research.

## Declaration of competing interest

The authors declare that they have no known competing financial interests or personal relationships that could have appeared to influence the work reported in this paper.

## Data Availability

The data supporting the findings of this study are available from the corresponding author upon reasonable request.
